# The Inositide Signaling Pathway As a Target for Treating Gastric Cancer and Colorectal Cancer

**DOI:** 10.3389/fphys.2016.00168

**Published:** 2016-05-09

**Authors:** Hong Jun Kim, Suk-young Lee, Sang Cheul Oh

**Affiliations:** Division of Oncology/Hematology, Department of Internal Medicine, College of Medicine, Korea UniversitySeoul, South Korea

**Keywords:** phosphoinositide, PI3K, mTOR, Akt, colorectal cancer, gastric cancer, targeted therapy

## Abstract

Gastric cancer and colorectal cancer are the leading cause of cancer mortality and have a dismal prognosis. The introduction of biological agents to treat these cancers has resulted in improved outcomes, and combination chemotherapy with targeted agents and conventional chemotherapeutic agents is regarded as standard therapy. Additional newly clarified mechanisms of oncogenesis and resistance to targeted agents require the development of new biologic agents. Aberrant activation of the inositide signaling pathway by a loss of function *PTEN* mutation or gain of function mutation/amplification of *PIK3CA* is an oncogenic mechanism in gastric cancer and colorectal cancer. Clinical trials with biologic agents that target the inositide signaling pathway are being performed to further improve treatment outcomes of patients with advanced gastric cancer and metastatic colorectal cancer (CRC). In this review we summarize the inositide signaling pathway, the targeted agents that inhibit abnormal activation of this signaling pathway and the clinical trials currently being performed in patients with advanced or metastatic gastric cancer and metastatic CRC using these targeted agents.

## Introduction

Phosphoinositides are ubiquitous signaling molecules in eukaryotes that are modified by phosphorylation at multiple positions on phosphatidylinositol (PtdIns). PtdIns is a membrane phospholipid containing a hydrophobic diacylglycerol and water soluble inositol ring. Phosphorylation at different positions on the inositol ring results in seven phosphoinositides (Sasaki et al., 2009). The phosphoinositide signaling system is essential for regulating cellular processes, including cell proliferation, vesicle transport, and cytoskeletal remodeling. Deregulation of phosphoinositide signaling by altering the genes encoding phosphoinositide-modifying enzymes leads to many diseases, such as cancers, inflammatory bowel disease, rheumatic disease, diabetes, and others (Bunney and Katan, 2010; Raimondi and Falasca, 2012).

In this article, we focus on the phosphoinositide signaling system and possible targeted therapies in gastric cancer (GC) and colorectal cancer (CRC).

## The phosphoinositide signaling system

**Figure 1:**
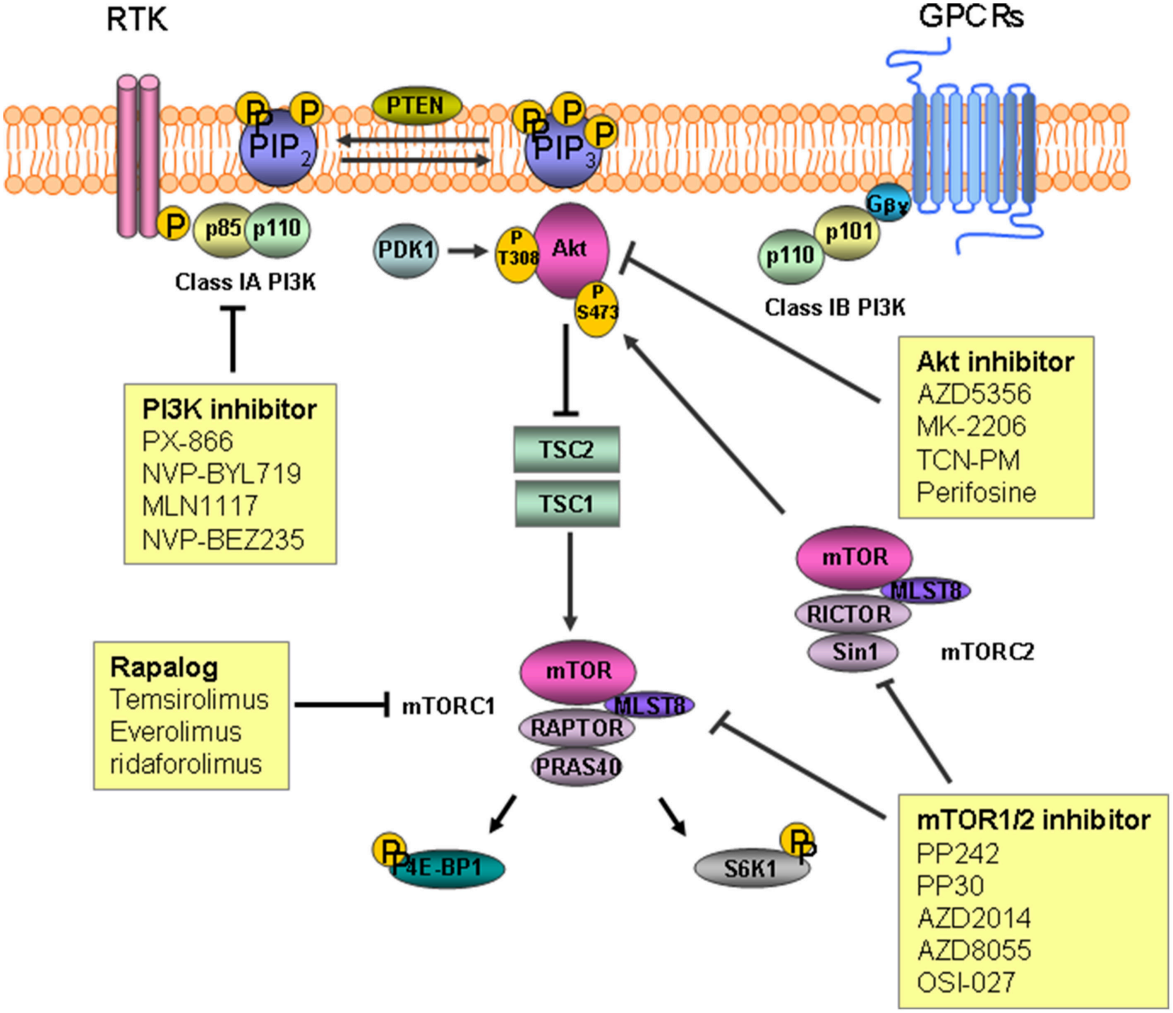
**The inositide signaling pathway and target agents used to treat gastric cancer and colorectal cancer**. Phosphatidylinosityo-4,5-bisphosphate [PtdIns(4,5)P_2_] is converted to phosphatidylinosityo-3,4,5-triphosphate [PtdIns(3,4,5)P_3_] by phosphoinositide 3-kinase (PI3K) as a result of phosphorylation. Akt/PKB is recruited to the plasma membrane followed by phosphorylation by mammalian target of rapamycin (mTOR) complex2 and 3-phosphoinositide-dependent protein kinase 1 (PDK1). Inactivation of tuberous sclerosis complex (TSC) 2 by Akt leads to degradation of TSC1/TSC2, which permits activation of mTOR. mTOR complex 1 phosphorylates downstream substrates S6 kinase 1(S6K1) and 4E-binding protein 1(4E-BP1), leading to mRNA translation initiation. RTK, receptor tyrosine kinase; GPCR, G-protein coupled receptor.

The parent PtdIns synthesized in the endoplasmic reticulum is phosphorylated into several different PtdIns monophosphates in the plasma membrane. The phosphorylated PtdIns4P further undergoes phosphorylation to PtdIns(4,5)P_2_ by PtdIns4P-5 kinases in the plasma membrane. Subsequent conversion to PtdIns (3,4,5)P_3_, a key lipid with signaling functions controlling cell growth and proliferation, is carried out by PI3K. The protein kinases, Akt/protein kinase B (PKB), 3-phosphoinositide-dependent protein kinase 1 (PDK1), and BTK cooperate to transduce different downstream signaling pathways. Dephosphorylation of PtdIns (3,4,5)P_3_ by PtdIns-3 phosphatase (PTEN) and PtdIns-5 phosphatase generate PtdIns (4,5)P_2_ and PtdIns (3,4)P_2_, respectively(Di Paolo and De Camilli, 2006; Engelman et al., 2006; Leslie et al., 2008; Ooms et al., 2009; Bunney and Katan, 2010). Phosphorylation of Akt/PKB on threonine 308 (Thr308) and serine 473 (Ser473) results in activation of the kinase and subsequent inactivation of tuberous sclerosis complex2 (TSC2) via phosphorylation. The inactivation of TSC2 leads to proteasomal degradation of the TSC1/TSC2 protein complex which in turn activates the mammalian target of rapamycin (mTOR) activation (Inoki et al., 2002; Potter et al., 2002; Figure 1).

## PI3K and PTEN

PI3K is a family of lipid kinases known to phosphorylate PtdIns(4,5)P_2_ at position 3 resulting in PtdIns(3,4,5)P_3._ PtdIns(3,4,5)P_3_ is a second messenger involved in regulating cell survival, proliferation, and growth, and its enhancement frequently leads to cancers (Engelman et al., 2006; Yuan and Cantley, 2008). PI3Ks are categorized into classes I–III based on their structures and substrate preference (Cantley, 2002). Class I PI3Ks are further grouped into two sub-families of class 1A and class1B according to their receptors. Class IA PI3Ks are heterodimeric proteins comprised of a regulatory subunit (p85α, p85β, or p55γ) and a catalytic subunit (p110α, p110β, or p110δ). Signaling from receptor tyrosine kinases (RTKs) is mediated through class IA PI3Ks by binding of the p85 regulatory subunit to phospho-tyrosine residues in the activated RTKs. The amino (N)-terminal p85-binding domain of the p110 catalytic subunit binds to the p85 regulatory subunit, and this interaction recruits the p85-p110 heterodimer to PtdIns(4,5)P_2_, which is a substrate located in the plasma membrane (Fruman et al., 1998; Engelman et al., 2006). Class IB PI3K is a heterodimer comprised of the p110 γ catalytic subunit and two regulatory subunits (p84 and p101). Class IB PI3K is activated by G-protein coupled receptors through interactions with the p101 regulatory subunit and the Gβγ subunit of the receptor (Stephens et al., 1997; Wymann et al., 2003). Class II PI3Ks consist of a p110-like catalytic subunit that preferentially phosphorylates PtdIns (Katso et al., 2001). Class III PI3Ks, or human vacuolar protein-sorting defective 34 (Vps34), regulates mTOR but little is known about their functions (Nobukuni et al., 2005). PTEN, which dephosphorylates PtdIns (3,4,5)P_3_, is a phosphatase that acts on phosphoinositides.

## Akt

Serine/threonine protein kinase Akt/PKB plays a role regulating cellular growth, survival, proliferation, and metabolism. Activation of Akt downstream of PI3K is mediated by phosphorylation with protein kinases. Following phosphorylation to PtdIns(3,4,5)P_3_ by PI3K, Akt/PKB is recruited to the plasma membrane enriched with PtdIns(3,4,5)P_3_ through its N-terminal pleckstrin homology (PH) domain. PDK1 then phosphorylates the recruited Akt at Thr308 on the central kinase catalytic domain (CAT). mTOR complex2 (mTORC2) is responsible for phosphorylation of the other Ser 473 site on the carboxyl terminal extension domain (EXT). Activated AKt/PKB then phosphorylates downstream substrates such as mTOR to control cellular functions (Alessi et al., 1997; Sabatini et al., 2005; Manning and Cantley, 2007; Gonzalez and McGraw, 2009).

## mTOR

mTOR is a 289-kDa serine/threonine kinase that plays a central role regulating cellular processes including cell growth and proliferation. mTOR exerts its effects by forming two distinct complexes distinguished according to the binding proteins mTORC1 and mTOR2. mTORC1 consists of mTOR, proline-rich Akt substrate 40 kDa, mammalian lethal with SEC13 protein8 (MLST8), and the regulatory-associated protein of mTOR. Activation of mTORC1 downstream of Akt/PKB subsequently phosphorylates substrates S6 kinase 1 (S6K1) and 4E-binding protein 1 (4E-BP1), which leads to initiation of mRNA translation and progression and stimulates protein synthesis. mTORC2 consists of mTOR, rapamycin-insensitive companion of mTOR, MLST8, and mammalian stress-activated protein kinase interacting protein 1. Activated mTORC2 phosphorylates Akt/PKB on Ser473, which is then fully activated with additional phosphorylation on Thr308 by PDK1 (Fingar et al., 2004; Sarbassov et al., 2005; Yang and Guan, 2007; Zoncu et al., 2011; Al-Batran et al., 2012).

## The role of phosphoinositide pathway dysregulation in the biology of stem cells

Tissue regeneration in the gut is ultimately regulated and sustained by intestinal stem cells (ISCs) that proliferate constantly and live long (Barker et al., 2010; Merlos-Suárez et al., 2011). In an animal study, ISCs were shown to initiate crypt expansion resulting in the formation of polyps, a precancerous neoplasm, but it is unclear which mutations in ISCs result in primary tumor initiation (He et al., 2007). Impaired bone morphogenic protein (BMP) signaling and activating mutations of Wnt signaling in ISCs can result in polyposis (Howe et al., 2001; Haramis et al., 2004; Sancho et al., 2004). There is evidence that PTEN and Akt could play crucial roles in the interaction between BMP and Wnt signals (He et al., 2007). Inactivation of PTEN was reported to cause Akt activation and activated Akt phosphorylates β-catenin, a main effector of the Wnt pathway, resulting in β-catenin's nuclear localization (Persad et al., 2001; He et al., 2007). In several organ systems including the intestine, nuclear translocation of β-catenin is considered to be crucial in activation of ISCs (Lowry et al., 2005). Bone morphogenetic protein4 (BMP4) treatment caused a substantial rise in PTEN levels in conjunction with the induction of differentiation and loss of tumorigenicity in CRC stem cells, but was not effective in stem cells harboring a *PI3KCA* mutation or with complete *PTEN* loss (Lombardo et al., 2011). These results show a coordination among BMP, PI3K/Akt, and Wnt signaling pathways in the biology of ISCs.

## Genetic/epigenetic aberrations of phosphoinositide signaling system in GI cancers

Mutations in the p110α, a catalytic subunit of class IA PI3K, are reported in 14–32% of patients with CRC (Samuels et al., 2004; Velho et al., 2005; Yuan and Cantley, 2008). Samuels et al. evaluated functional effects of the mutation of *PIK3CA* in CRC by inactivation of *PIK3CA* mutation in CRC cell lines. They reported *PIK3CA* mutations facilitate tumor invasion and attenuate apoptosis (Samuels et al., 2005). Studies on the prognosis of patients with CRC harboring *PIK3CA* mutations have reported controversial results, and the impact of the mutation has been regarded as insignificant (Cathomas, 2014). In GC, the *PIK3CA* mutation is reported in 4–25% (Samuels et al., 2004; Li et al., 2005; Velho et al., 2005). A study concerning the role of amplification of *PIK3CA* gene in GC reported a high frequency (67%) of amplification in GC and that amplification of *PIK3CA* is associated with poor prognosis (Shi et al., 2012) (Table 1).

**Table 1:** **Genetic aberrations and their effects on prognosis**.

**Cancer type (references)**	**Aberration of *PI3KCA* or *PTEN***	**Effect on prognosis**
**GASTRIC CANCER**		
Shi et al., 2012	Amplification of *PI3KCA*	Poor survival
**COLON CANCER**		
Iida et al., 2012	Mutation or methylation of *PI3KCA*	Poor survival
Phipps et al., 2013	Mutation of *PI3KCA*	Poor survival
Eklöf et al., 2013	Mutation of *PI3KCA*	No association
	Loss of *PTEN*	No association
Mouradov et al., 2013	Mutation of *PI3KCA*	No association

## Preclinical studies of phosphoinositide pathway

Several studies have demonstrated that the phosphoinositide pathway is associated with proliferation, apoptosis, and metastasis. A preclinical study demonstrated activation of Akt1 promoted cell survival by showing inhibition of Akt1 phosphorylation and subsequent inhibited cell growth with LY294002, a PI3K inhibitor, in GC. In this study, dominant negative Akt also showed inhibition of proliferation and induction of cell cycle arrest in GC cells (Han et al., 2008). Another study showed upregulated *PIK3CA* expression is associated with lymph node metastasis in GC (Liu et al., 2010). Xing et al. investigated the effects of LY294002 on invasiveness with a GC mouse xenograft model. They found that LY294002 inhibited tumor growth and promoted apoptosis (Xing et al., 2009). The role of *PIK3CA* mutations was also demonstrated in CRC by showing inhibition of growth in *PIK3CA* mutant CRC cell lines by treatment with LY294002 (Samuels et al., 2005).

## Next druggable target candidate

PI3K expression of metastatic tumors in CRC is higher than that of primary tumors (Zhu et al., 2012), suggesting that PI3K might contribute to the progression and distant metastasis of CRC as in other advanced stage cancers. As activating *PIK3CA* mutations are observed in up to 20% of CRCs, many PI3K inhibitors have been studied (DeVita et al., 2008). Three types of PI3K inhibitors are now available for targeted therapy of solid tumors, such as Pan-class I inhibitors, isoform specific PI3K inhibitors, and dual PI3K/mTOR inhibitors (Vadas et al., 2011; Martini et al., 2013).

## Pan- class I inhibitors

Pan-class I inhibitors are active against all p110 isoforms. These inhibitors include quecertin, the first non-specific PI3K inhibitor, wortmannin, LY294002, PX-866, NVP-BKM120, ZSTK474, BKM120, GDC0941, XL147, and BAY80-6946 (Singh et al., 2015). Wortmannin is a potent and specific PI3K inhibitor that binds covalently to Lys802 on the catalytic subunit of p110α and to Lys883 on the p110γ subunit (Powis et al., 1994; Wymann et al., 1996; Walker et al., 2000). Despite the potent inhibitory effect of wortmannin against PI3K, its short half-life, biological instability, and toxicity limits its clinical application (Yuan and Cantley, 2008). PX-866 is a biologically stable semisynthetic viridian derivative of wortmannin that shows good pharmacokinetics and has a prolonged inhibitory effect on PI3K (Ihle et al., 2004). A recent multicenter phase I trial of PX-866 reported tolerable toxicity and prolonged stable disease in patients with untreatable solid tumors including GC and CRC (Hong et al., 2012). BKM120 is an oral pyrimidine-derived inhibitor that targets class I PI3Ks but not class III PI3K or mTOR (Pecchi et al., 2010). In a phase I clinical trial, BKM120 was tolerated and demonstrated preliminary activity against advanced cancers (Bendell et al., 2012).

## Isoform-specific PI3K inhibitors

Isoform-specific inhibitors were produced with the hope of taking advantage of the superior efficacy of pan PI3K inhibitors without the unwanted side effects. These inhibitors include NVP-BYL719, CAL-101, GSK2636771, and MLN1117 (INK1117). NVP-BYL719 is an α-specific PI3K inhibitor derived from the 2-aminothiazole class (Furet et al., 2013). A phase I clinical trial of BYL719 in combination with the heat shock protein (HSP) 90 inhibitor AUY922 in patients with advanced gastric cancer has been completed recently (NCT01613950). A phase I clinical trial of NVP-BYL719 including patients with metastatic CRC bearing *PIK3CA* mutations was performed (Juric et al., 2012). In this study, NVP-BYL719 had tolerable side effects and acceptable efficacy. INK1117 is a potent, α-selective PI3K inhibitor with good oral bioavailability that inhibits proliferation of tumor cell lines carrying *PIK3CA* mutations (Jessen et al., 2011). Results of a phase I clinical trial performed on patients with advanced solid malignancies including GC treated with MLN1117 were recently reported. The antitumor activity of this agent was demonstrated by showing an objective response in patients with breast cancer and GC (NCT01449370; Juric et al., 2015). The β-selective PI3K inhibitor GSK2636771 was developed based on the preclinical observation that selective depletion of a PI3K isoform expression reduces tumorigenesis in *PTEN*-deficient tumors (Rivero and Hardwicke, 2012). As loss of the *PTEN* is prevalent in CRCs, further assessment of GSK2636771 in patients with CRC is necessary. An *in vitro* study suggested that activation of PI3K plays a crucial role in resistance against a BRAF inhibitor in patients with CRC carrying the *BRAF* mutation (Mao et al., 2013).

## Dual PI3K/mTOR inhibitors

Pan PI3K/mTOR inhibitors block the activities of both PI3Ks and mTOR kinases by competitively binding to the ATP-binding sites. Because mTOR is structurally related to PI3Ks, ATP-competitive compounds inhibit these two kinases with equivalent potency. In comparison with mTORC1 inhibitor, dual PI3K/mTOR inhibitors could overcome loss of mTORC1-dependent negative feedback on PI3K signaling (Roper et al., 2011). mTORC1 blockade might be attenuated by resultant mTORC2-mediated activation of Akt by phosphorylation at Ser473 (Roper et al., 2011). NVP-BEZ235, NVP-BGT226, VS-5884, PI-103, XL765, GDC-0980, and PF-05212384 are dual Pan PI3K/mTOR inhibitors. In a preclinical study using a genetically engineered mouse model carrying wild-type *PIK3CA* with CRC, NVP-BEZ235 induced tumor regression (Roper et al., 2011). Considering the relatively low prevalence of the activating *PIK3CA* mutation (up to 20%), further studies should be performed on the efficacy of NVP-BEZ235 in patients with wild-type *PIK3CA*.

## Akt inhibitors

Akt/PKB is an essential protein kinase comprised of three isoforms, such as Akt1 (PKBα), Akt2 (PKBβ), and Akt3 (PKBγ). The Akt structure consists of three conserved domains, including an N-terminal PH domain, a CAT domain, and a C-terminal EXT domain. Akt inhibitors are classified depending on the mechanism of action, such as compounds that compete for the ATP-binding site, allosteric inhibitors, agents targeting the PH domain, and pseudosubstrate inhibitors (Kumar and Madison, 2005). Among these groups, allosteric inhibitors and ATP mimetics are the two main groups that have been investigated for clinical application. Several Akt inhibitors are currently being studied for GC, including AZD5363, MK-2206, triciribine phosphate monohydrate (TCN-PM), perifosine, GDC-0068, and GSK690693. AZD5363 is a potent ATP-competitive inhibitor of Akt and a novel pyrrolopyrimidine-derived compound that acts against all Akt isoforms (Luke et al., 2011; Davies et al., 2012). MK-2206 is an allosteric inhibitor that binds to the PH and kinase domains, which blocks transport of Akt to the membrane and it activation (Hirai et al., 2010). Results of a phase II study of MK-2206 in patients with GC have been recently released. Patients with GC and gastroesophageal junction cancer who progressed to first-line treatment were administrated MK-2206. The primary endpoint of median overall survival (OS) was 5.1 months (m) [95% confidence interval (CI), 3.7–9.4 m] and did not meet the study efficacy endpoint of 6.5 m (Ramanathan et al., 2015). In a phase I study, MK-2206 was well tolerated with evidence of Akt blockade (Yap et al., 2011). Subsequently, two phase II studies were planned. TCN-PM exerts its effect by targeting the PH domain and blocking translocation of Akt to the plasma membrane (Berndt et al., 2010). A phase I trial of TCN-PM in patients with solid tumors including gastroesophageal cancer carrying increased phosphorylated Akt (p-Akt) reported modest decreases in tumor p-AKT after TCN-PM monotherapy treatment (Garrett et al., 2011).

## mTOR inhibitors

Two kinds of mTOR inhibitors called rapalogs and mTORC1/2 inhibitors have been reported and categorized by their specificity for mTOR complexes. Rapamycin is the first mTOR inhibitor discovered. Formation of an inhibitory complex upon binding of rapamycin to the intracellular receptor FK506 binding protein 12 (FKBP12) leads to binding of the complex to the FKB12-rapamycin binding domain at the C-terminus of TOR proteins, which subsequently prevents mTOR from signaling downstream targets (Kunz and Hall, 1993; Chen et al., 1995; Choi et al., 1996; Zhou et al., 2010). Temsirolimus, everolimus, and ridaforolimus are rapalogs that have been studied for treating GC. Temsirolimus is a dihydroxymethyl propionic acid ester of rapamycin that inhibits phosphorylation of S6K1 and 4E-BP1 mediated by mTOR (Rini, 2008). A phase I trial to determine the pharmacokinetics of temsirolimus in patients with advanced cancers including GC showed an anti-tumor effect of this agent (Hidalgo et al., 2006). Everolimus is an oral formula of a rapamycin analog. A phase II trial was conducted to investigate the efficacy of everolimus combined with capecitabine in patients with refractory GC. The primary endpoint of overall response rate was 10.6% (Lee et al., 2013). In the phase III GRNITE-1 study, patients with refractory GC who had undergone at least one prior systemic chemotherapeutic trial were randomized to receive everolimus with best supportive care (BSC) or placebo plus BSC. However, the primary endpoint of significantly improved OS was not observed in the everolimus group (median OS: 5.4 vs. 4.3 m; hazard ratio, 0.9; 95% CI, 0.75–1.08; *p* = 0.124; Ohtsu et al., 2013). Despite the failure of meeting the primary endpoint, significant improvement in progression free survival was observed, and one of causes of the failure might have been the effect of a salvage therapy after progression to the treatment in clinical trial. mTOR1/2 inhibitors exert their effects against mTORC1 and mTORC2 at nanomolar concentrations without inhibiting other kinases. These new-generation ATP-competitive mTOR inhibitors include PP242, PP30, AZD2014, AZD8055, and OSI-027 (Feldman et al., 2009; Zhou et al., 2010).

## Dual targeted strategy for the RAS/MEK/ERK and PI3K/Akt/mTOR pathways

Treating patients with metastatic CRC using biologic agents combined with chemotherapeutic agents has become standard therapy. Although the epidermal growth factor receptor monoclonal antibody has brought improved outcomes in those with wild-type *RAS* (Fakih, 2015), the acquired resistance to the monoclonal antibody, even in patients with CRC carrying wild-type *RAS*, has been a treatment issue possibly due to crosstalk between signaling pathways (Chong and Jänne, 2013). Several studies have suggested the possibility of a combined therapy targeting the RAS/MEK/ERK and PI3K/Akt/mTOR pathways (Yu et al., 2008). A dual PI3K/mTOR inhibitor combined with the MEK inhibitor selumetinib produces growth-suppressive effects in patient-derived xenografts from CRC with mutated *RAS* (Migliardi et al., 2012). Shimizu et al. retrospectively analyzed clinical outcomes of 236 patients who received the dual targeted strategy involving the RAS/MEK/ERK and PI3K/AKT/mTOR pathways (Shimizu et al., 2012). Although the study focused on drug safety rather than efficacy, the clinical outcomes suggested that the dual targeted strategy is more effective compared with monotherapy in properly selected patients (Shimizu et al., 2012).

## Potential pharmacodynamic markers of inhibitors

Since PI3K pathway inhibitors have a role in physiological glucose metabolism, estimating insulin resistance could be a good pharmacodynamic marker (Engelman et al., 2006; Luo et al., 2006).

Assessment of phosphorylation of Akt at Thr308 and Ser473, 4E-BP1 at Ser65 and Thr70, ribosomal protein S6 (RPS6) at Ser240 and Ser244, or PRAS40 could offer potential as pharmacodynamic markers (Rodon et al., 2013). Quantification of these molecular pharmacodynamic biomarkers have been performed in not only tumor tissues but also surrogate tissues, like peripheral blood mononuclear cells, platelet-enriched plasma, skin, and hair, and should be feasible in the clinic (Jimeno et al., 2010; Biondo et al., 2011; Rodon et al., 2013). However, these methods have that limitation that increased RAS.RAF/ERK/mTORC1 activity could adjust phosphorylation of RPS6, 4E-BP1, or PRAS40 (Manning and Cantley, 2007).

## Conclusion

Phosphoinositides are versatile and indispensable for regulating various cellular functions. The phosphoinositide signaling system can become deregulated occasionally because of mutations in genes encoding kinases or phosphatases, and consequently, becomes integral to carcinogenesis and progression of GC and CRC. Therefore, the phosphoinositide pathway is an important target for the development of anticancer drugs.

Several key steps are required to develop optimal targeted agents. A novel approach can be formulated by using tumor genotypic and molecular biologic analyses. Patients should be properly selected based on preclinical data and well standardized predictive markers for combined targeted therapy to be effective. Avoiding bias in well-planned clinical trials is also important for the success of targeted therapy.

## Author contributions

SL designed, wrote and edited the manuscript. HK wrote and edited the manuscript. SO supervised and edited the manuscript.

### Conflict of interest statement

The authors declare that the research was conducted in the absence of any commercial or financial relationships that could be construed as a potential conflict of interest.
